# Factors affecting low fetal fraction in fetal screening with cell-free DNA in pregnant women: a systematic review and meta-analysis

**DOI:** 10.1186/s12884-022-05224-7

**Published:** 2022-12-08

**Authors:** Sanaz Mousavi, Ziba Shokri, Parvin Bastani, Morteza Ghojazadeh, Sevda Riahifar, Hooman Nateghian

**Affiliations:** 1 Department of Gynecology and Obstetrics, Al-Zahra Hospital, Faculty of Medicine, Tabriz University of Medical Sciences, Tabriz, Iran; 2 Research Center for Evidence‑Based Medicine, Iranian EBM Centre: A Joanna Briggs Institute Affiliated Group, Tabriz University of Medical Sciences, Tabriz, Iran; 3 Department of Biostatistics, Faculty of Public Health, Iran University of Medical Sciences, Tehran, Iran

**Keywords:** Low fetal fraction, Cell-free DNA, Fetal soft marker, Fetal screening

## Abstract

**Background:**

Cell-Free DNA (cfDNA) is a non-invasive perinatal test (NIPT) used to assess fetal anomalies. The ability to detect fetal chromosomal aneuploidies is directly related to a sample’s fetal to total DNA fraction, known as the fetal fraction (FF). The minimum FF is considered 4%, and the test result below 4% is uncertain due to low fetal fraction (LFF). This study aimed to conduct a systematic review and a meta-analysis to determine the possible factors affecting LFF in cfDNA testing for fetal screening.

**Methods:**

PubMed, Web of Science, Google Scholar, Since Direct, Scopus, CINHAL, Cochrane Library, and Persian databases, including Scientific Information Database, Irandoc, and Magiran were searched for studies investigating factors affecting LFF in cfDNA testing from 2000 until the end of 2021. Gathered data were analyzed using Comprehensive Meta-Analysis (CMA) software version 3.3.070. The quality of the included studies was assessed using the Joanna Briggs Institute Critical Appraisal of Cohort Studies tool.

**Results:**

Thirteen articles related to the topic were included, and seven related articles were reviewed for meta-analysis. The other six were reviewed qualitatively. Four factors were identified that might have a potential effect on the LFF, of which only gestational age had a significant association with LFF (Pooled mean difference= -1.111, SE = 0.515, 95% CI= -2.121, -0.101, (*P*-value < 0.05)). Maternal age (*P*-value = 0.573), maternal weight (*P*-value = 0.113), and Body Mass Index (*P*-value = 0.104) had no statically significant effect. The effect size was pooled by mean difference and 95% confidence interval.

**Conclusion:**

Lower gestational age is significantly associated with LFF. Thus, this factor can be considered when interpreting prenatal cfDNA screening tests.

**Supplementary Information:**

The online version contains supplementary material available at 10.1186/s12884-022-05224-7.

## Introduction

Cell-Free DNA (cfDNA) is a non-invasive perinatal test (NIPT) used to assess fetal anomalies such as aneuploidies. cfDNA is assessed using maternal blood sampling [1]. Cell-free DNA originates from fetal trophoblasts [2], and its fragment size is smaller than that of maternal DNA [1]. Approximately 11 to 13.4% of Cell-Free DNA in maternal blood is of embryonic origin [3], which appears in the mother’s blood within the 5 to 7 weeks of pregnancy. The amount of cfDNA increases with gestational age and tends to decrease after delivery. It would be cleared within two hours from the mother’s blood after delivery [4]. Compared to standard invasive screening techniques such as Chorionic Villus Sampling (CVS) and amniocentesis, Cell-Free DNA testing is non-invasive, easy to perform, and has no risk of miscarriage [5]. It is recommended to perform Cell-Free DNA in cases of age over 35, positive aneuploidy screening in the presence of increased nuchal translucency, abnormal ultrasound findings, and positive personal or familial history of aneuploidy [6]. cfDNA is a mixture of maternal and fetal cfDNA, and the ability to detect fetal chromosomal aneuploidies is directly related to the fetal to total DNA fraction of a sample. This ratio is the fetal fraction (FF) [7]. If the FF is too small, any abnormalities in the fetal cfDNA will be masked by the overwhelming proportion of euploid maternal cfDNA, thereby making their detection impractical [8]. Recent studies have consistently shown that the average FF is around 10–15% but can range up to 30% or more. The minimum FF is considered 4%, and the test result below 4% is uncertain [9]. Overestimated FF would lead to false-negative results, while underestimated FF may cause the rejection of suitable samples. According to studies, several factors such as body mass index (BMI), gestational age, twin pregnancy, and pregnancy biomarkers in maternal serum affect the amount of Cell-Free DNA and interrupt the interpretation of FF. Pregnancy screening tests impose huge costs on the people and the government, and Incorrect interpretation of results may lead to potentially inappropriate medical decisions [10]. Given the importance of cfDNA tests in prenatal screening and ambiguity around factors affecting LFF, we decided to conduct a systematic and meta-analysis study on studies retrieved through a rigorous search and selection process to detect the factors causing LFF that lead to false results.

## Methods

The review was conducted according to the PRISMA statement [11]. In addition, a protocol was designed before initiating the search.

### Search strategy

Two independent reviewers conducted a comprehensive literature search in the following databases: Medline, Science Direct, Scopus, Web of Science, Springer, Cochrane, Oxford Journals, Willey online library, Microsoft academic search, Mosby, EBSCO, Karger, and CINHAL. As for Persian databases, Magiran, IranDoc, IranMedex, and SID were searched. Gray literature and studies presented at conferences were also reviewed. An attempt was made to reach out to people working in the field for more information on published and unpublished studies. In addition, relevant references in selected studies were examined thoroughly to find related studies that were not found in our search. The following MeSH and free keywords were used; “cell-free DNA,“ “low fetal fraction,“ “fetal soft marker,“ AND “Down syndrome screen.“ In addition to the above keywords, synonyms, abbreviated symbols, and other free keywords were used. Persian keywords were used to retrieve Persian studies. In the case of multiple publications of one article, the most updated and comprehensive one was adopted. The search strategy designed for this study can be accessed in the supplementary file.

The selected studies in our study followed these criteria: 1) cross-sectional and cohort studies, 2) studies presented in congresses, 3) studies conducted from 2000 to the end of 2021, 4) only English and Persian studies, 5) Studies reporting the diagnostic value of cfDNA testing. Only factors related to FF below 4% were included in this review. Studies with inappropriate design, conducted before 2000, in languages other than English and Persian, examining unrelated subjects, not evaluating the association of studied parameters, and studies that were reviews, meta-analyses, or systematic reviews were excluded.

### Data extraction and synthesis

The quality of the included studies was verified using the Joanna Briggs Institute Critical Appraisal of Cohort Studies tool. Results of the quality appraisal are accessible in the supplementary file. This tool presents 11 questions evaluating different points in the study, which should be answered with “No,“ “unclear,“ “not applicable,“ or “Yes.“ Each Yes response corresponds to one point, so the tool score ranges from 0 to 11, reported in percent. Studies totaling 70% or more were considered low risk of bias; 50–69% were of medium risk of bias, and 50% or lower were considered to have a high risk of bias. To classify the studies, two reviewers performed the classification independently. Disagreements were resolved through discussion until a consensus was reached. Studies not meeting sufficient quality were discarded after a thorough examination. For each eligible study, the following characteristics were collected: first author, year of publication, the country in which the study was conducted, study type, number of sufficient fetal fractions (SFF) and low fetal fractions (LFF), and mean or median(SD-maximum and minimum) based on the affecting factor (gestational age, maternal age, BMI and maternal body weight).

### Statistical analysis

Extracted data were summarized in Excel from Microsoft office 2013. Endnote X5 was used to organize the studies and discard the duplicates. The mean difference between the two groups was selected as the effect size index. The I-Squared index was used to examine the heterogeneity between studies. I-Squared values less than 50% were considered homogeneous. In the presence of heterogeneity (*p* ≤ 0.1), the random-effects model was used; otherwise, the fixed-effects model was used. I^2^ ≥ 50% was considered as high heterogeneity. Funnel diagrams and Egger tests were used to investigate diffusion bias. A probability value of less than 0.05 was considered significant. All analyzes were performed using Comprehensive Meta-Analysis (CMA) software version 3.3.070.

## Results

### Study selection and study characteristics

**Fig. 1:**
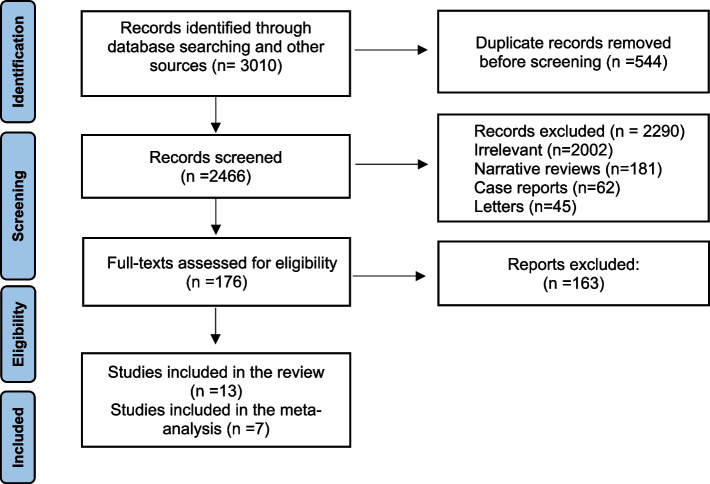
The Preferred Reporting Items for Systematic Reviews and Meta-Analyses (PRISMA) flow diagram for search and selection of the relevant studies

Based on the search study stated above, 3010 studies were identified. Five hundred forty four studies were discarded due to duplication. The title and abstract parts of the remaining 2466 studies were reviewed, of which 2290 studies were excluded due to irrelevance. Discarded studies included 62 case-report studies, 45 letters, 181 review studies, and 2002 unrelated studies. The full text of the remaining 176 studies was reviewed, of which 163 were excluded due to irrelevance to the study. Finally, 13 studies were eligible and included in the study. The PRISMA flow chart related to the search process is shown in Fig. 1. During the review of articles, four influential factors were identified to have enough data for meta-analysis: (1) gestational age, (2) maternal age, (3) maternal weight (4) BMI. Other identified factors were reviewed qualitatively.

The characteristics of all the included studies are summarized in Table 1 based on the effective factors on FF.

**Table 1:** Summary characteristics of the included studies for meta-analyses

**Study**	**Year**	**Country**	**Study type**	**Number of LFF**	**Number of SFF**	**Mean of GA (LFF)**	**SD (min–max) Of GA (LFF)**	**Mean of GA (SFF)**	**SD (min–max) Of GA (SFF)**	**Mean of Maternal Age (LFF)**	**SD (min–max) Of Maternal Age (LFF)**
**Burns W [**12**]**	**2017**	**USA**	**Retrospective cohort**	***18***	***2875 ***	**14.3**	**(-, -) 3.78**	**13.4**	**(-, -) 3.33**	**37.3**	**5.90**
**Dabi Y [**13**]**	**2018**	**France**	**Prospective cohort**	***58***	***295 ***	**12.6**	**(27.5- 11.1)**	**-**	**(-, -) -**	**34**	**(35.7–40.1)**
**Krishna I [**14**]**	**2016**	**USA**	**Retrospective cohort**	***22***	***348 ***	**16.4**	**(-, -) 4.2**	**17**	**(-, -) 5.5**	**35**	**(22–46)**
**Miltoft CB [**15**]**	**2019**	**Denmark**	**Prospective cohort**	***10***	***321 ***	**-**	**(-, -) -**	**-**	**(-, -) -**	**33.3**	**4.2**
**Nakamura N [**16**]**	**2020**	**Japan**	**Retrospective cohort**	***23***	***2628 ***	**12.1**	**(14.7– 10.0) 1.3**	**12.7**	**(18.4 – 10.0) 1.6**	**38.6**	**2(34.8–43.4)**
**Wang E [**3**]**	**2013**	**USA**	**Prospective cohort**	***357***	***22384 ***	**13.9**	**(-, -) 4.55**	**15.79**	**(-, -) 4.55**	**-**	**-**
**Zhao Q [**17**] **	**2019**	**China**	**Prospective cohort**	***42***	***2202 ***	**14.43**	**(-, -) 4.20**	**16.95 / 16.57**	**(26 – 12) 2.89**	**-**	**-**

**Study**	**Mean of Maternal Age (SFF)**	**SD (min–max) Of Maternal Age (SFF)**	**Mean of BMI (LFF)**	**SD (min–max) of BMI (LFF)**	**Mean of BMI (SFF)**	**SD (min–max) of BMI (SFF)**	**Mean of Maternal Weight (LFF)**	**SD (min–max) of Maternal Weight (LFF)**	**Mean of MW (SFF)**	**SD (min–max) of Maternal Weight (SFF)**
**Burns W [**12**] **	**39.2**	**11.6**	**34.6**	**(-, -) 8.90**	**26.4**	**(-, -) 6.25**	**-**	**(-, -) -**	**-**	**(-, -) -**
**Dabi Y [**13**] **	**34**	**(29.6–37.1)**	**29**	**(32.9 – 22.3) -**	**23.7**	**(25.9—20.7) -**	**77**	**(90.3 -60) -**	**64**	**(70—56) -**
**Krishna I [**14**]**	**35**	**(14–46)**	**36.5**	**(54.6- 22.9) -**	**29.1**	**(54.9 – 17.3) -**	**-**	**(-, -) -**	**-**	**(-, -) -**
**Miltoft CB [**15**]**	**32.7**	**4.2**	**19.5**	**(24.2 –20.3) -**	**21.8**	**(24.2 – 20.3) -**	**-**	**(-, -) -**	**-**	**(-, -) -**
**Nakamura N [**16**]**	**39**	**2.6(24.8–49.1)**	**20.9**	**(29.1 – 15.7) 2.8**	**20.8**	**(34.8 – 14.5) 2.6**	**-**	**(-, -) -**	**-**	**(-, -) -**
**Wang E [**3**] **	**-**	**-**	**-**	**(-, -) -**	**-**	**(-, -) -**	**103**	**(-284 32) -**	**73**	**(-, -) 8.66**
**Zhao Q [**17**]**		**-**	**23.1/23.7**	**(39.7 – 15.6) 3.6**	**23.1**	**(-, -) 3.58**	**-**	**(-, -) -**	**-**	**(-, -) -**

LFF* Low Fetal Fraction,* SFF* Sufficient Fetal Fraction,* GA* Gestational Age,* MA* Maternal Age,* MW* Maternal Weight,* BMI* Body Mass Index,* SD *Standard Deviation*

### Qualitative review

Six studies [8, 18–21] were not included in the meta-analysis due to insufficient quantitative data following the study’s objectives. They were included in the study for qualitative review (Table 2). Marwan (2017) [21] reported that FF < 4% was more seen in greater BMI. Ashoor et al. (2013) [18] investigated the relationship between the FF of the cfDNA test and maternal and fetal characteristics. FF < 4% was reported more in pregnant women with high weight gain. In the Caucasian race, reporting of FF < 4% decreased with increasing crown rump length (CRL). Based on Kinnings et al. (2015) [8], the incidence of FF < 4% increases with high BMI and doing the test at earlier gestational age. Kuhlmann-Capek et al. (2019) [19] study indicated that obesity and consumption of two or more medications (regardless of the medication type) are associated with a high incidence of FF < 4%. The studied drugs included aspirin, Plavix, heparin, antibiotics, chemotherapy, antivirals, anti-diabetic and anti-thyroid drugs. Lee et al. (2018) [20] studied the cell-free DNA in singleton IVF pregnancies. LFF was more incident in IVF pregnancies than in spontaneous pregnancies and also was linked to elevated BMI. Rolnik et al. (2018) [22] investigated the association between BMI and Cell-free DNA test failure and concluded that patients with high BMI had LFF.

**Table 2:** Summary Characteristics of included studies for qualitative syntheses

**N**	**Author**	**Year**	**Country**	**Study type**	**Objectives**	**Number/characteristics of Pts**	**Lower limit of FF** **FF technique**	**Results**
**1**	Ali M [21]	2017	USA	Retrospective Cohort Study	Evaluation of pregnancy complications associated with low FF‌ in NIPT test	256 Pregnant women	4%	This study investigated the relationship between low FF and pregnancy complications. Low FF incident was higher in people with high BMI, and perinatal complications were higher in people with low FF.
**2**	Ashoor G [18]	2013	UK	Retrospective Cohort Study	Evaluating the effective factors on FF and maternal and fetal characteristics	1949 singleton pregnancies within 11 to 13 weeks of pregnancy	4% Harmony	FF < 4% was more frequent in women with higher weight. FF < 4% decreased by the increase of CRL in the Caucasian race.
**3**	Kinnings S [8]	2015	USA	Retrospective cohort study	Study of effective factors on low FF in NIPT test of pregnant women	140,377 pregnant women	3.7% MPS	There was a direct relationship between gestational age and FF < 4% There was an inverse relationship between maternal BMI and FF < 4%
**4**	Kuhlmann-Capek M [19]	2019	USA	Retrospective Cohort Study	Investigating the relationship between drug use in early pregnancy and FF in NIPT test	1051 pregnant women with singletons, of which 400 had positive drug history (divided into two groups:1)only one drug 2)two or more drugs) and 651 women had a negative history	4% SNP	Obesity and the use of two or more drugs (regardless of the type of drug used) were associated with a higher risk ratio of FF, less than 4%
**5**	Lee T [20]	2018	AUS	Retrospective Cohort Study	The relationship between IVF pregnancy and FF rate in singleton pregnant women	A total of 5,625 pregnant women with singleton over 10 weeks, including 4,633 normal pregnancies and 992 IVF pregnancies	4% Harmony	Increased BMI and IVF were recognized as an effective factor of FF < 4%, but the type of IVF method (ICSI-Standard-fresh cycle-frozen cycle) was not associated with low FF.
**6**	Rolnik D [22]	2018	AUS	Cross-Sectional Study	The effect of BMI on the increase of FF index with increasing age and failure of NIPT test	14,233 pregnant women with singleton over ten weeks of age were included in the study, of which 8583 were tested by method A and the rest by method B.	Platform, A(4%) digital analysis of selected regions for chromosome analysis and single nucleotide polymorphism analysis platform B(2%) next-generation sequencing and massive parallel sequencing for aneuploidy screening	On both platforms, the mean FF rate was lower in the group with higher BMI, and as a result, the test failure rate was higher in this group. Inconclusive tests and consequently low FF was more common among people with higher BMI.

### Meta-analysis

Four factors, including maternal age, gestational age, maternal weight, and BMI, had enough numerical data for meta-analysis of potential effectors of LFF (FF < 4%).

**Fig. 2:**
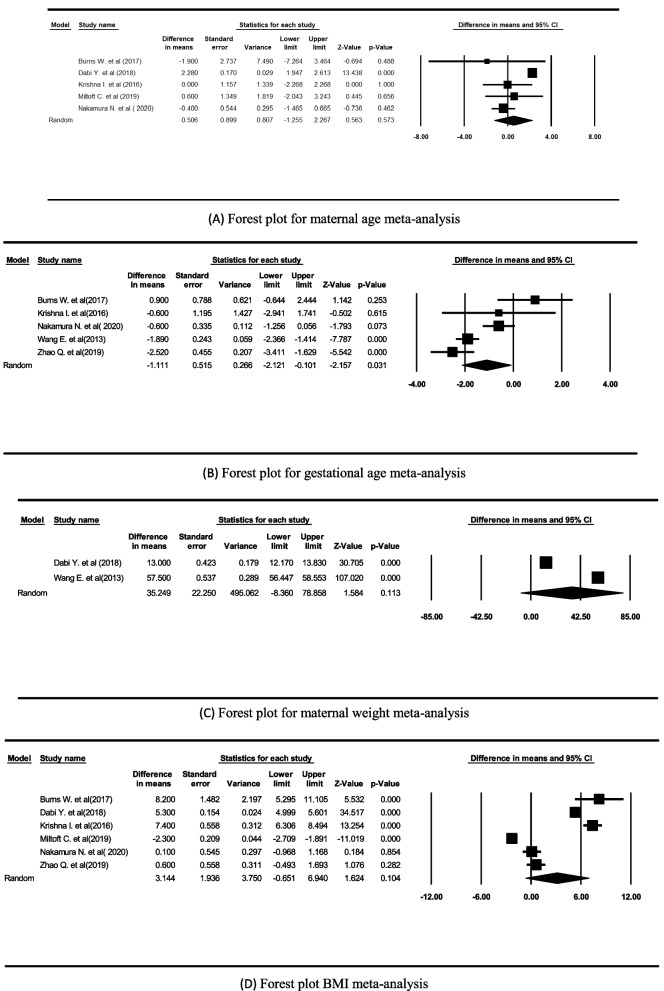
Effect of maternal age (**A**), gestational age (**B**), maternal weight (**C**), and maternal BMI (**D**) on low fetal fraction in cfDNA pregnancy tests

To estimate the difference between the merged means between low fetal fraction (LFF) and sufficient fetal fraction (SFF), the means and medians of LFF and SFF were entered into the meta-analysis from each study. The forest plot for the size of the integrated effect from the selected studies is shown in Fig. 2.

#### Maternal age

Five studies [12–14, 16] were included in the meta-analysis for maternal age. There was no statistically significant association between maternal age and LFF (*P*-value = 0.573). Heterogeneity between studies was significant (Q = 28.278, *P* < 0.001, I^2^ = 85.855). Pooled mean difference = 0.506, SE = 0.899, (95% CI = -1.255, 2.282) (Fig. 2a).

#### Gestational age

Meta-analysis of five relevant studies [3, 12, 14, 16, 17] demonstrated that gestational age is significantly related to LFF (*P*-value = 0.031). Heterogeneity was significant (Q = 24.662, *P*-value < 0.001, I2 = 83.781). Pooled mean difference= -1.111, SE = 0.515, (95% CI= -2.121, -0.101) (Fig. 2b).

#### Maternal weight

Two studies [3, 13] were included to analyze maternal weight and LFF association. The results showed no significant correlation between maternal weight and LFF (*P*-value = 0.113). Heterogeneity was significant (Q = 4231.919, *P*-value < 0.001, I2 = 99.975. Pooled mean difference = 35.249, SE = 22.250, (95% CI = -8.360, 78.858) (Fig. 2c).

#### BMI

Meta-analysis of six relevant studies [12–15, 17] showed no significant association between BMI and LFF (*P*-value = 0.104). Heterogeneity was significant (Q = 982.078, *P*-value < 0.001, I2 = 99.491). Pooled mean difference = 3.144, SE = 1.936, (95% CI = -0.651, 6.940) (Fig. 2d).

## Publication bias

**Fig. 3:**
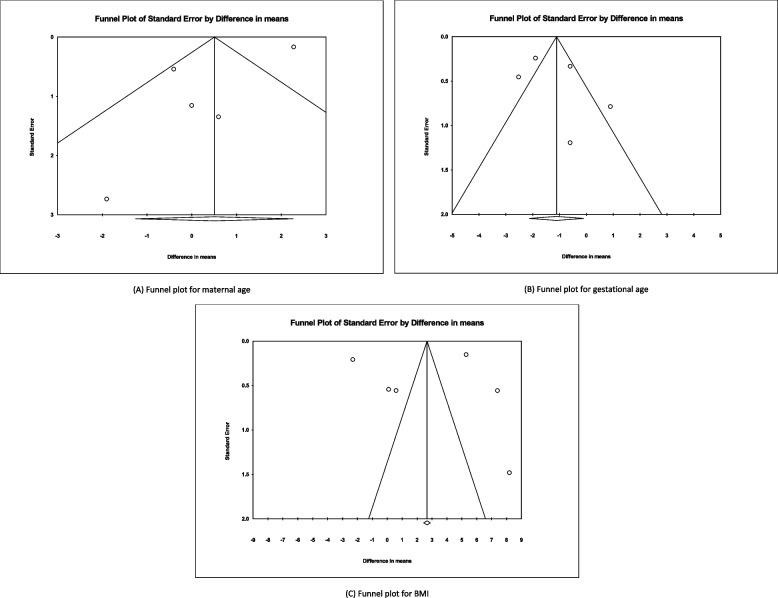
Begg’s funnel plot for studies included in the meta-analysis of maternal age (**A**), gestational age (**B**), and BMI (**C**). Each open circle represents one the studies in our meta-analysis. The Standard Error for each study is plotted versus difference in means. The circles are distributed equally around the solid vertical line with a solid diamond at the bottom, representing the overall effect in this study

Begg’s funnel plots and Egger’s test were applied to assess the potential publication bias (Fig. 3).

No publication bias was detected for any of the conducted meta-analyses. Since only two studies were available for the maternal weight variable according to the results, a publication bias assessment was impossible (maternal age *P*-value = 0.124, gestational age *P*-value = 0.447, BMI *P*-value = 0.903).

## Discussion

cfDNA testing is one of the NIPT tests used to assess aneuploidies in the fetus and is widely used for routine invasive tests [1]. The mother’s bloodstream cDNA consists of maternal and embryonic types. For the test results to be conclusive, there should be a certain minimum amount of embryonic cfDNA. This value might vary based on the technique and kit used in different laboratories; however, a 4% threshold of the FF is considered sufficient [9]. This study is the first meta-analysis that evaluates factors affecting the reduction of FF ratios to less than 4% in the mother’s blood.

Factors that could alter the FF ratio are generally divided into three categories: maternal, fetal-placental, and experimental [23].

Maternal factors mentioned in the literature include maternal age, maternal weight, gestational age, race [14, 15], in vitro fertilization (IVF) [20], consumption of certain drugs [12, 19], and maternal diseases, especially autoimmune diseases [24]. Some studies have reported free β-subunit of human chorionic gonadotropin (free β-hCG) and serum pregnancy-associated plasma protein (PAPP-A) levels to be positively correlated with FF [25, 26].

Several fetal-placental factors other than gestational age have also been reported to alter FF. these include crown-rump length [18, 25], gender [26], twin pregnancies [27, 28], preterm birth [29, 30], and aneuploidies [31, 32]. Experimental causes related to lab procedures include cfDNA fragment size, cfDNA concentration, library concentration, and uniquely mapped reads [33].

We identified four potential factors with enough data for meta-analysis (maternal age, gestational age, maternal weight, and BMI) that could cause LFF. The meta-analysis identified an association between gestational age and LFF; however, there was no significant relationship between the other three named factors and LFF. When the substantial heterogeneity in 95% prediction intervals was accounted for, the results indicated that the association between LFF and maternal age, maternal weight, and BMI became insignificant. These results do not necessarily indicate that there is no impact of named factors on LFF; however, the results do indicate that there is still substantial uncertainty about the significance of the association.

Five studies [3, 12, 14, 16, 17] discussed gestational age’s effect on LFF [3, 14, 16, 18, 22]. The mean gestational age at Burns et al. (2017) [12] study was reported as 14.3 weeks, and at Krishna et al. (2016) [14] was 16.4 weeks, which was irrelevant with LFF. In Nakamura et al. (2020) [16] study, the gestational age was 12.1 weeks, Wang et al. (2013) [3] was 13.9 weeks, and Zhao et al. (2019) [17] was 14.43 weeks, and in these three studies, it was related to a LFF.

Five studies [12–14, 16] discussed maternal age’s effect on the LFF. The mean maternal age in Burns et al. (2017) [12] study was 37.3 years, and in Miltoft et al. (2020) [15] was 33.3 years. Krishna et al. (2016) [14] reported 22 to 46 years, while Nakamura et al. (2020) [16] reported 34.8 to 43.4 years, All irrelevant to LFF. The mean maternal age of Dabi et al. (2018) [13] was 34 and related to LFF.

Two studies [3, 13] discussed the effect of maternal weight on the LFF [3–11, 19]. In Dabi et al. (2018) [13], the maternal weight range was (60-90.3 kg), and the mean was 77 kg. The mean maternal weight in Wang et al. (2013) [3] study was 103 kg, which was related to the LFF in these two studies.

Maternal weight meta-analysis heterogeneity might be due to different gestational ages chosen for inclusion criteria within the studies. The gestational age range in Dabi et al. (2018) [13] study was 11.1 to 27.5 weeks, whereas it was 11.1 to 40.43 weeks in Wang et al. (2013) [3] study. Since there is a constant increase in maternal weight as the pregnancy progresses, the difference in the gestational age range might be the clinical cause of heterogeneity. Of all the studies, Wang et al. (2013) [3] had the broadest range of gestational age and the largest sample size (22,384 pregnant women); we speculate that the uneven distribution of gestational age within the selected studies for gestational age meta-analysis might be the cause of heterogeneity.

Six studies [12–15, 17] discussed the BMI effect on LFF [13, 14, 16, 18, 19, 22]. The mean BMI was 34.6 in Burns et al.  (2017) [12] study and 29 in Dabi et al. (2018) [13] study and 36.5 in Krishna et al. (2016) [14] study, and 19.5 in Miltoft et al. (2020) [15] study and 23.1 at Zhao et al. (2019) [17] study and it was related to the LFF in these five studies. The mean BMI was 20.9 in Nakamura et al. (2020) [16] study, which was irrelevant.

Other implications for LFF in prenatal care have been suggested as well. In a systematic review by Scheffer et al. published in 2021, LFF in cfDNA testing was associated with adverse pregnancy outcomes, particularly pregnancy-related hypertensive disorders, preterm delivery, and impaired fetal growth [34]. Shree et al. concluded that in mothers with BMI < 30 kg/m2, those with hypertensive disorders of pregnancy have lower fetal fraction; however, obesity affected LFF in such a way that it could not be used for predicting hypertension in obese individuals [35].

Further and more extensive studies are needed to investigate possible factors affecting FF thoroughly. In addition, comprehensive strategies can be developed to reduce the chance of encountering LFF by designing tailored and beneficial interventions targeting modifiable factors.

## Limitations and strengths

This review had some limitations that are worth mentioning: the relatively small number of articles included in this review, only studies between the years 2000 and 2021 were examined, only the most prevalent factors with potential effects were included in this study, and finally, only English and Persian articles were examined.

Along with its limitations, this study had noteworthy strength points. The topmost advantage of this systematic review was the low risk of subjective data selection. Predefined criteria guided the search process, quality assessment, and data synthesis and two independent reviewers performed those using well-established tools. This study was the first comprehensive systematic review and meta-analysis that evaluated the factors affecting LFF in fetal screening with Cell-Free DNA in pregnant women.

## Conclusion

A number of factors were reported to have a potential effect on the amount of FF such as maternal age, maternal BMI, maternal weight, and gestational age. However, after meta-analyses of the mentioned factors, only gestational age significantly affected the amount of FF in the cfDNA tests. Lower gestational age is significantly associated with LFF. Thus, this factor needs to be considered in interpreting the prenatal cfDNA screening tests to make a more accurate interpretation.

## Supplementary Information

**Additional file 1**.

## Data Availability

All data generated or analyzed during this study are included in this published article and its supplementary information files.
