# Supplementary material for: Factors affecting low fetal fraction in fetal screening with cell-free DNA in pregnant women: a systematic review and meta-analysis
Source: BMC Pregnancy Childbirth. 2022 Dec 8;22:918. doi: 10.1186/s12884-022-05224-7 (PMC9733315; doi:10.1186/s12884-022-05224-7)
Supplement: Supplementary file 1 — Additional file 1. [file 12884_2022_5224_MOESM1_ESM.docx]

**Search strategy**

('pregnancy'/mj OR 'child bearing':ti OR 'childbearing':ti OR 'gestation':ti OR 'gravidity':ti OR 'intrauterine pregnancy':ti OR 'labor presentation':ti OR 'labour presentation':ti OR 'pregnancy':ti OR 'pregnancy maintenance':ti OR 'pregnancy trimesters':ti OR 'trisomy'/mj OR 'trisomic':ti,ab OR 'trisomy':ti,ab OR 'fetal soft marker':ti,ab OR 'fetal fraction'/mj) AND ('cell free dna'/mj OR 'noninvasive prenatal testing'/exp OR 'cell-free dna (cfdna) screening':ti OR 'cell-free dna (cfdna) testing':ti OR 'cfdna screening':ti OR 'cfdna testing':ti OR 'non-invasive prenatal screening':ti OR 'non-invasive prenatal testing':ti OR 'noninvasive prenatal cell-free dna screening':ti OR 'noninvasive prenatal screening':ti OR 'noninvasive prenatal testing':ti OR 'prenatal cell free dna (cfdna) testing':ti OR 'prenatal cell-free dna (cfdna) screening':ti OR 'prenatal cell-free dna screening':ti OR 'prenatal cell-free dna testing':ti OR 'prenatal cfdna screening':ti OR 'prenatal cfdna testing':ti)

( TITLE ( "cell free dna" OR "noninvasive prenatal testing" OR "cell-free dna (cfdna) screening" OR "cell-free dna (cfdna) testing" OR "cfdna screening" OR "cfdna testing" OR "non-invasive prenatal screening" OR "non-invasive prenatal testing" OR "noninvasive prenatal cell-free dna screening" OR "noninvasive prenatal screening" OR "noninvasive prenatal testing" OR "prenatal cell free dna (cfdna) testing" OR "prenatal cell-free dna (cfdna) screening" OR "prenatal cell-free dna screening" OR "prenatal cell-free dna testing" OR "prenatal cfdna screening" OR "prenatal cfdna testing" ) ) AND ( TITLE-ABS-KEY ( "pregnancy" OR "child bearing" OR "childbearing" OR "gestation" OR "gravidity" OR "intrauterine pregnancy" OR "labor presentation" OR "labour presentation" OR "pregnancy" OR "pregnancy maintenance" OR "pregnancy trimesters" OR "trisomy" OR "trisomic" OR "trisomy" OR "fetal soft marker" OR "fetal fraction" ) )

| study  **Results of JBI critical appraisal checklist for cohort studies** | Q1 | Q2 | Q3 | Q4 | Q5 | Q6 | Q7 | Q8 | Q9 | Q10 | Q11 | %yes | Risk |
| --- | --- | --- | --- | --- | --- | --- | --- | --- | --- | --- | --- | --- | --- |
| Burns.w 2017 | yes | yes | yes | yes | yes | yes | yes | yes | yes | yes | yes | 100% | low |
| Dabi.Y 2018 | yes | yes | yes | yes | yes | yes | yes | yes | yes | yes | yes | 100% | low |
| Krishna.I 2016 | yes | yes | yes | yes | yes | yes | yes | yes | yes | yes | yes | 100% | low |
| Miltoft.C 2019 | yes | yes | yes | yes | yes | yes | yes | yes | yes | yes | yes | 100% | low |
| Nakamura.N 2020 | yes | yes | yes | yes | yes | yes | yes | yes | yes | yes | yes | 100% | low |
| Wang.E 2013 | yes | yes | yes | yes | yes | yes | yes | yes | yes | yes | yes | 100% | low |
| Zhao.Q 2019 | yes | yes | yes | yes | yes | yes | yes | yes | yes | yes | yes | 100% | low |

Q.1: Were the two groups similar and recruited from the same population? Q.2: Were the exposures measured similarly to assign people to both exposed and unexposed groups? Q.3: Was the exposure measured in a valid and reliable way? Q.4: Were confounding factors identified? Q.5: Were strategies to deal with confounding factors stated? Q.6: Were the groups/participants free of the outcome at the start of the study (or at the moment of exposure)? Q.7: Were the outcomes measured in a valid and reliable way? Q.8: Was the follow-up time reported and sufficient to be long enough for outcomes to occur? Q.9: Was follow up complete, and if not, were the reasons to loss to follow up described and explored? Q.10: Were strategies to address incomplete follow up utilized? Q.11: Was appropriate statistical analysis used?

List of article excluded at the full-text level

Reasons:

1-Most of articles were excluded due to lack of review of Fetal fraction below 4%. In majority of reviewed articles, Fetal fraction was between 8-10 %.

2- Failure to examine relevant variables

3-Incomplete information

4- Inappropriate study plan

1. Kudryavtseva, E., Kovalev, V., Baranov, I., Kanivets, I., Kievskaya, Y. K., & Korostelev, S. Low Fetal Fraction of Cell-free DNA Identified by Non-invasive Prenatal DNA Testing: Possible Causes, Clinical Significance, and Tactics.
2. Farina, A., LeShane, E. S., Lambert-Messerlian, G. M., Canick, J. A., Lee, T., Neveux, L. M., Palomaki, G. E., & Bianchi, D. W. (2003). Evaluation of cell-free fetal DNA as a second-trimester maternal serum marker of Down syndrome pregnancy. *Clinical chemistry*, *49*(2), 239-242.
3. Wataganara, T., Chen, A. Y., LeShane, E. S., Sullivan, L. M., Borgatta, L., Bianchi, D. W., & Johnson, K. L. (2004). Cell-free fetal DNA levels in maternal plasma after elective first-trimester termination of pregnancy. *Fertility and sterility*, *81*(3), 638-644.
4. Bauer, M., Hutterer, G., Eder, M., Majer, S., LeShane, E., Johnson, K. L., Peter, I., Bianchi, D. W., & Pertl, B. (2006). A prospective analysis of cell‐free fetal DNA concentration in maternal plasma as an indicator for adverse pregnancy outcome. *Prenatal Diagnosis: Published in Affiliation With the International Society for Prenatal Diagnosis*, *26*(9), 831-836.
5. Gil, M. M., Quezada, M. S., Bregant, B., Ferraro, M., & Nicolaides, K. H. (2013). Implementation of maternal blood cell-free DNA testing in early screening for aneuploidies. *Ultrasound Obstet Gynecol*, *42*(1), 34-40.
6. Lau, T. K., Jiang, F. M., Chan, M. K., Zhang, H. Y., Lo, P. S. S., & Wang, W. (2013). Non-invasive prenatal screening of fetal Down syndrome by maternal plasma DNA sequencing in twin pregnancies. *Journal of Maternal-Fetal & Neonatal Medicine*, *26*(4), 434-437.
7. Poon, L., Musci, T., Song, K., Syngelaki, A., & Nicolaides, K. (2013). Maternal plasma cell-free fetal and maternal DNA at 11-13 weeks' gestation: relation to fetal and maternal characteristics and pregnancy outcomes. *Fetal diagnosis and therapy*, *33*(4), 215-223.
8. Verweij, E. J., Jacobsson, B., van Scheltema, P. A., de Boer, M. A., Hoffer, M. J., Hollemon, D., Westgren, M., Song, K., & Oepkes, D. (2013). European non-invasive trisomy evaluation (EU-NITE) study: a multicenter prospective cohort study for non-invasive fetal trisomy 21 testing. *Prenat Diagn*, *33*(10), 996-1001.
9. Christiansen, S. C., Vanky, E., Klungland, H., Stafne, S. N., Mørkved, S., Salvesen, K., Sæther, M., & Carlsen, S. M. (2014). The effect of exercise and metformin treatment on circulating free DNA in pregnancy. *Placenta*, *35*(12), 989-993.
10. Comas, C., Echevarria, M., Prats, P., Rodríguez, I., & Serra, B. (2014). OP 03.02: Initial experience of non‐invasive prenatal testing with cell‐free DNA: factors influencing fetal fraction. *Ultrasound in Obstetrics & Gynecology*, *44*(S1), 69-69.
11. del Mar Gil, M., Quezada, M. S., Bregant, B., Syngelaki, A., & Nicolaides, K. H. (2014). Cell-free DNA analysis for trisomy risk assessment in first-trimester twin pregnancies. *Fetal Diagn Ther*, *35*(3), 204-21
12. Grömminger, S., Yagmur, E., Erkan, S., Nagy, S., Schöck, U., Bonnet, J., Smerdka, P., Ehrich, M., Wegner, R. D., Hofmann, W., & Stumm, M. (2014). Fetal aneuploidy detection by cell-free DNA sequencing for multiple pregnancies and quality issues with vanishing twins [Article]. *Journal of Clinical Medicine*, *3*(3), 679-692.
13. Hofmann, W., Grömminger, S., Schöck, U., Bonnet, J., & Smerdka, P. (2014). Non-invasive prenatal testing (NIPT): Laboratory experiences of PrenaTest® [Conference Abstract]. *Medizinische Genetik*, *26*(1), 194.
14. Petersen, O. B., Vogel, I., Ekelund, C., Hyett, J., & Tabor, A. (2014). Potential diagnostic consequences of applying non-invasive prenatal testing: population-based study from a country with existing first-trimester screening. *Ultrasound Obstet Gynecol*, *43*(3), 265-27
15. Rava, R. P., Srinivasan, A., Sehnert, A. J., & Bianchi, D. W. (2014). Circulating fetal cell-free DNA fractions differ in autosomal aneuploidies and monosomy X [Article]. *Clinical Chemistry*, *60*(1), 243-250.
16. Schlütter, J. M., Hatt, L., Bach, C., Kirkegaard, I., Kølvraa, S., & Uldbjerg, N. (2014). The cell-free fetal DNA fraction in maternal blood decreases after physical activity. *Prenat Diagn*, *34*(4), 341-344.
17. Struble, C. A., Syngelaki, A., Oliphant, A., Song, K., & Nicolaides, K. H. (2014). Fetal fraction estimate in twin pregnancies using directed cell-free DNA analysis. *Fetal Diagn Ther*, *35*(3), 199-203.
18. Tarquini, F., Di Renzo, G. C., Picchiassi, E., Centra, M., Pennacchi, L., Galeone, F., Bini, V., & Coata, G. (2014). 205: Maternal smoking and the amount of cell free fetal DNA in maternal plasma during the first trimester of pregnancy. *American Journal of Obstetrics & Gynecology*, *210*(1), S111-S112.
19. Zhou, Q., Pan, L., Chen, S., Chen, F., Hwang, R., Yang, X., Wang, W., Jiang, J., Xu, J., Huang, H., & Xu, C. (2014). Clinical application of noninvasive prenatal testing for the detection of trisomies 21, 18, and 13: A hospital experience [Article]. *Prenatal Diagnosis*, *34*(11), 1061-106
20. Alberti, A., Salomon, L. J., Le Lorc'h, M., Couloux, A., Bussières, L., Goupil, S., Malan, V., Pelletier, E., Hyon, C., Vialard, F., Rozenberg, P., Bouhanna, P., Oury, J. F., Schmitz, T., Romana, S., Weissenbach, J., Vekemans, M., & Ville, Y. (2015). Non-invasive prenatal testing for trisomy 21 based on analysis of cell-free fetal DNA circulating in the maternal plasma [Article]. *Prenatal Diagnosis*, *35*(5), 471-47
21. Bevilacqua, E., Gil, M. M., Nicolaides, K. H., Ordonez, E., Cirigliano, V., Dierickx, H., Willems, P. J., & Jani, J. C. (2015). Performance of screening for aneuploidies by cell-free DNA analysis of maternal blood in twin pregnancies. *Ultrasound in Obstetrics & Gynecology*, *45*(1), 61-66.
22. Comas, C., Echevarria, M., Rodríguez, M. A., Prats, P., Rodríguez, I., & Serra, B. (2015). Initial experience with non-invasive prenatal testing of cell-free DNA for major chromosomal anomalies in a clinical setting. *J Matern Fetal Neonatal Med*, *28*(10), 1196-1201.
23. Fiorentino, F., Spinella, F., Bono, S., Pizzuti, F., Mariano, M., Polverari, A., Duca, S., Cottone, G., Nuccitelli, A., Teresa Sessa, M., & Baldi, M. (2015). Feasibility of noninvasive prenatal testing for common fetal aneuploidies in maternal serum with low levels circulating fetal cell-free DNA fraction [Conference Abstract]. *Prenatal Diagnosis*, *35*, 1.
24. Larion, S., Warsof, S., Romary, L., Mlynarczyk, M., & Abuhamad, A. (2015). Three year clinical experience with noninvasive prenatal testing in 3000 high risk cases in the United States [Conference Abstract]. *Prenatal Diagnosis*, *35*, 59
25. Norem, C., Obolensky, E., Bijesse, E., Turocy, J., Blumberg, B., Fehlen-Quizon, P., Krieger, R., & Wohlferd, M. (2015). Non-invasive prenatal screening for trisomies-2 years experience in a large Health Maintenance Organization (HMO) [Conference Abstract]. *Prenatal Diagnosis*, *35*, 62
26. Norton, M., Musci, T., & Wapner, R. (2015). Relationship between 1st trimester fetal fraction of cell-free DNA from maternal plasma and preeclampsia in a large general pregnancy population. *American Journal of Obstetrics and Gynecology*, *212*(1), S188-S188.
27. Palomaki, G. E., Kloza, E. M., Lambert-Messerlian, G. M., van den Boom, D., Ehrich, M., Deciu, C., Bombard, A. T., & Haddow, J. E. (2015). Circulating cell free DNA testing: are some test failures informative? *Prenat Diagn*, *35*(3), 289-293.
28. Quezada, M. S., Gil, M. M., Francisco, C., Orosz, G., & Nicolaides, K. H. (2015). Screening for trisomies 21, 18 and 13 by cell-free DNA analysis of maternal blood at 10-11 weeks' gestation and the combined test at 11-13 weeks. *Ultrasound in Obstetrics & Gynecology*, *45*(1), 36-41.
29. Radoi, V. E., Bohiltea, C. L., Bohiltea, R. E., & Albu, D. N. (2015). Cell free fetal DNA testing in maternal blood of Romanian pregnant women. *Iran J Reprod Med*, *13*(10), 623-626.
30. Sago, H., Sekizawa, A., & Japan, N. C. (2015). Nationwide demonstration project of next-generation sequencing of cell-free DNA in maternal plasma in Japan: 1-year experience. *Prenatal Diagnosis*, *35*(4), 331-336
31. Zhang, H., Gao, Y., Jiang, F., Fu, M., Yuan, Y., Guo, Y., Zhu, Z., Lin, M., Liu, Q., Tian, Z., Chen, F., Lau, T. K., Zhao, L., Yi, X., Yin, Y., & Wang, W. (2015). Non-invasive prenatal testing for trisomies 21, 18 and 13: clinical experience from 146,958 pregnancies [Article]. *Ultrasound in obstetrics & gynecology : the official journal of the International Society of Ultrasound in Obstetrics and Gynecology*, *45*(5), 530-538
32. Aziz, A., Van Arsdale, A., Klugman, S., & Wright, R. L. (2016). Detection of Fetal Fraction During Noninvasive Prenatal Screening (NIPS) in HIV-Infected Pregnant Women. *Obstetrics and Gynecology*, *127*, 9S-9S.
33. Koumbaris, G., Kypri, E., Tsangaras, K., Achilleos, A., Mina, P., Neofytou, M., Velissariou, V., Christopoulou, G., Kallikas, I., González-Liñán, A., Benusiene, E., Latos-Bielenska, A., Marek, P., Santana, A., Nagy, N., Széll, M., Laudanski, P., Papageorgiou, E. A., Ioannides, M., & Patsalis, P. C. (2016). Cell-Free DNA analysis of targeted genomic regions in maternal plasma for non-invasive prenatal testing of trisomy 21, trisomy 18, trisomy 13, and fetal sex [Article]. *Clinical Chemistry*, *62*(6), 848-855.
34. Mnyani, C. N., Nicolaou, E., & Bister, S. (2016). The value and role of non-invasive prenatal testing in a select South African population [Article]. *South African Medical Journal*, *106*(10), 1047-1050.
35. Revello, R., Sarno, L., Ispas, A., Akolekar, R., & Nicolaides, K. H. (2016). Screening for trisomies by cell-free DNA testing of maternal blood: consequences of a failed result. *Ultrasound Obstet Gynecol*, *47*(6), 698-704
36. Sarno, L., Revello, R., Hanson, E., Akolekar, R., & Nicolaides, K. H. (2016). Prospective first-trimester screening for trisomies by cell-free DNA testing of maternal blood in twin pregnancy. *Ultrasound Obstet Gynecol*, *47*(6), 705-711.
37. Suzumori, N., Ebara, T., Yamada, T., Samura, O., Yotsumoto, J., Nishiyama, M., Miura, K., Sawai, H., Murotsuki, J., Kitagawa, M., Kamei, Y., Masuzaki, H., Hirahara, F., Saldivar, J. S., Dharajiya, N., Sago, H., Sekizawa, A., & Japan, N. C. (2016). Fetal cell-free DNA fraction in maternal plasma is affected by fetal trisomy. *Journal of Human Genetics*, *61*(7), 647-652.
38. Benn, P. (2017). The Significance of Test Failures in Noninvasive Prenatal Screening for Fetal Aneuploidy Using Cell-free DNA. *Journal of Fetal Medicine*, *4*(1), 13-18.
39. Ershova, E., Sergeeva, V., Klimenko, M., Avetisova, K., Klimenko, P., Kostyuk, E., Veiko, N., Veiko, R., Izevskaya, V., Kutsev, S., & Kostyuk, S. (2017). Circulating cell-free DNA concentration and DNase I activity of peripheral blood plasma change in case of pregnancy with intrauterine growth restriction compared to normal pregnancy. *Biomed Rep*, *7*(4), 319-324.
40. Flöck, A., Tu, N. C., Rüland, A., Holzgreve, W., Gembruch, U., & Geipel, A. (2017). Non-invasive prenatal testing (NIPT): Europe’s first multicenter post-market clinical follow-up study validating the quality in clinical routine [Article]. *Archives of Gynecology and Obstetrics*, *296*(5), 923-928.
41. Kadam, P., Venkatawamy, E., Tayal, T., Nampoorthiri, S., Andrew, C., Kabra, M., Bagga, R., Gowda, M., Batra, M., Hegde, S., Kaul, A., Subramanian, J. G., Lingaiah, S., Akhtar, R., Kidangan, F., Chandran, R., Kiran, C., Ravi Kumar, G. R., Ramprasad, V. L., . . . Verma, I. C. (2017). Noninvasive prenatal testing-an Indian study [Conference Abstract]. *Prenatal Diagnosis*, *37*, 48-49
42. Silver, R. M., Myatt, L., Hauth, J. C., Leveno, K. J., Peaceman, A. M., Ramin, S. M., Samuels, P., Saade, G., Sorokin, Y., Clifton, R. G., & Reddy, U. M. (2017). Cell-Free Total and Fetal DNA in First Trimester Maternal Serum and Subsequent Development of Preeclampsia. *Am J Perinatol*, *34*(2), 191-198.
43. Williams, J., Gupta, M., Rad, S., Ozimek, J., Ratousi, D., Farivar, S., Pisarska, M., & Schreck, R. (2017). Positive and negative predictive values of cell-free DNA for noninvasive prenatal testing [Conference Abstract]. *Prenatal Diagnosis*, *37*, 41-42.
44. Hartwig, T. S., Ambye, L., Werge, L., Weiergang, M. K., Nørgaard, P., Sørensen, S., & Jørgensen, F. S. (2018). Non-Invasive Prenatal Testing (NIPT) in pregnancies with trisomy 21, 18 and 13 performed in a public setting - factors of importance for correct interpretation of results. *Eur J Obstet Gynecol Reprod Biol*, *226*, 35-39.
45. Le Conte, G., Letourneau, A., Jani, J., Kleinfinger, P., Lohmann, L., Costa, J. M., & Benachi, A. (2018). [Cell-free fetal DNA analysis in maternal plasma as a screening test for trisomy 21 in twin pregnancies]. *Gynecol Obstet Fertil Senol*, *46*(7-8), 580-586.
46. Li, M., Riming, L., Zhe, W., Hong, W., Xiaofei, H., Lina, C., Zhengfang, Z., Xuebo, W., Ning, W., Chengming, S., Qing-Qing, H., Hailiang, L., & Hui, W. (2018). Non-invasive prenatal screening for chromosome 21, 18, and 13 aneuploidies in a mixed risk factors pregnancy population [Article]. *Clinical and Experimental Obstetrics and Gynecology*, *45*(4), 523-528.
47. Morano, D., Rossi, S., Lapucci, C., Pittalis, M. C., & Farina, A. (2018). Cell-Free DNA (cfDNA) Fetal Fraction in Early- and Late-Onset Fetal Growth Restriction. *Mol Diagn Ther*, *22*(5), 613-619.
48. Rafaeli-Yehudai, T., Imterat, M., Douvdevani, A., Tirosh, D., Benshalom-Tirosh, N., Mastrolia, S. A., Beer-Weisel, R., Klaitman, V., Riff, R., Greenbaum, S., Alioshin, A., Rodavsky Hanegbi, G., Loverro, G., Catalano, M. R., & Erez, O. (2018). Maternal total cell-free DNA in preeclampsia and fetal growth restriction: Evidence of differences in maternal response to abnormal implantation. *PLoS One*, *13*(7), e0200360.
49. Rizzo, G., Mappa, I., Alessio, A., Veglia, M., Quarto, A., & Piscicelli, C. (2018). P20. 03: Relationship between uterine artery pulsatility index and fetal fraction cell‐free DNA in maternal plasma at 11+ 0 to 13+ 6 weeks of gestation. *Ultrasound in Obstetrics & Gynecology*, *52*, 191-191.
50. Suzumori, N., Sekizawa, A., Ebara, T., Samura, O., Sasaki, A., Akaishi, R., Wada, S., Hamanoue, H., Hirahara, F., Izumi, H., Sawai, H., Nakamura, H., Yamada, T., Miura, K., Masuzaki, H., Yamashita, T., Okai, T., Kamei, Y., Namba, A., . . . Sago, H. (2018). Fetal cell-free DNA fraction in maternal plasma for the prediction of hypertensive disorders of pregnancy. *Eur J Obstet Gynecol Reprod Biol*, *224*, 165-169.
51. Takeda, E., Suzumori, N., Kumagai, K., Inuzuka, S., Oseto, K., Ohigashi, Y., Yotsumoto, J., Miyake, H., & Sugiura-Ogasawara, M. (2018). Performance and outcomes of noninvasive prenatal testing for twin pregnancies in Japan. *J Obstet Gynaecol Res*, *44*(10), 1909-1914.
52. Xuan, L., Yin, K. L., Xia, Y., Mao, Y., Shen, J., Zhu, Y., Xue, Y., Feng, S. D., Liu, H., & Liang, B. (2018). Data analysis of non-invasive prenatal testing based on special loci in cell-free fetal DNA [Article]. *Chinese Journal of Medical Genetics*, *35*(1), 56-59
53. Yang, J. X., Qi, Y. M., Hou, Y. P., Guo, F. F., Peng, H. S., Wang, D. M., Haoxin, O. Y., Wang, Y. X., Huang, H. J., & Yin, A. H. (2018). Performance of non-invasive prenatal testing for trisomies 21 and 18 in twin pregnancies. *Molecular Cytogenetics*, *11*, Article 47.
54. Zhang, B., Pan, L., Wang, H., Liu, J., Lu, B., Chen, Y., Long, W., & Yu, B. (2018). [Performance of prenatal screening by non-invasive cell-free fetal DNA testing for women with various indications]. *Zhonghua Yi Xue Yi Chuan Xue Za Zhi*, *35*(1), 51-55.
55. Zhang, B., Shen, C., Wang, H., Cai, Z., Lu, B., Zhang, X., Yu, B., & Wang, T. (2018). Preliminary analysis of the cause for the failure of non-invasive prenatal testing using cell-free fetal DNA derived from peripheral maternal blood [Article]. *Chinese Journal of Medical Genetics*, *35*(3), 329-333.
56. Bender, W. R., Koelper, N. C., Sammel, M. D., & Dugoff, L. (2019). Association of Fetal Fraction of Cell-Free DNA and Hypertensive Disorders of Pregnancy. *Am J Perinatol*, *36*(3), 311-316.
57. Carrara, J., Vivanti, A., Jani, J. C., Demain, A., Costa, J. M., & Benachi, A. (2019). Usefulness and reliability of cell free fetal DNA screening for main trisomies in case of atypical profile on first trimester maternal serum screening. *J Transl Med*, *17*(1), 398.
58. Chen, Y., Yu, Q., Mao, X., Lei, W., He, M., & Lu, W. (2019). Noninvasive prenatal testing for chromosome aneuploidies and subchromosomal microdeletions/microduplications in a cohort of 42,910 single pregnancies with different clinical features [Article]. *Human Genomics*, *13*(1)
59. Clapp, M. A., Shook, L. L., Roberts, P. A., Goldfarb, I. T., & Bernstein, S. N. (2019). Clinical implications of low fetal fraction in pregnancies with negative cell free DNA testing. *American Journal of Obstetrics and Gynecology*, *220*(1), S163-S163.
60. Dyr, B., Boomer, T., Almasri, E. A., Wardrop, J. L., Rafalko, J., Chibuk, J., & McCullough, R. M. (2019). y A new era in aneuploidy screening: cfDNA testing in > 30,000 multifetal gestations: Experience at one clinical laboratory. *Plos One*, *14*(8), Article e0220979.
61. Lund, I. C. B., Vestergaard, E. M., Lildballe, D., Becher, N., Petersen, O. B., & Vogel, I. (2019). Clinical experience of noninvasive prenatal testing for fetal trisomies 21, 18 and 13 in high and intermediate-risk pregnancies after public combined first trimester screening [Conference Abstract]. *European Journal of Human Genetics*, *26*, 128-129.
62. Minarik, G., Landlova, D., Lukackova, R., Izsakova, A., Mojzisikova, I., Sekelska, M., Valentinova, L., & Krizan, P. (2019). Prospective validation study of utilization of Trisomy test for noninvasive prenatal testing of common trisomies [Conference Abstract]. *European Journal of Human Genetics*, *26*, 146.
63. Qiao, L., Zhang, Q., Liang, Y., Gao, A., Ding, Y., Zhao, N., Zhang, W., Li, H., Lu, Y., & Wang, T. (2019). Sequencing of short cfDNA fragments in NIPT improves fetal fraction with higher maternal BMI and early gestational age. *Am J Transl Res*, *11*(7), 4450-4459.
64. Sekelska, M., Izsakova, A., Kubosova, K., Tilandyova, P., Csekes, E., Kuchova, Z., Hyblova, M., Harsanyova, M., Kucharik, M., Budis, J., Szemes, T., & Minarik, G. (2019). Result of prospective validation of the trisomy Test® for the detection of chromosomal trisomies [Article]. *Diagnostics*, *9*(4).
65. Yin, Y., Zhu, H., Qian, Y., Jin, J., Mei, J., & Dong, M. (2019). Noninvasive prenatal screening for twin pregnancy: an analysis of 2057 cases [Article]. *Zhejiang da xue xue bao. Yi xue ban = Journal of Zhejiang University. Medical sciences*, *48*(4), 403-408.
66. Basaran, S., Has, R., Kalelioglu, I. H., Karaman, B., Kirgiz, M., Dehgan, T., Satkin, B. N., Sivrikoz, T. S., & Yuksel, A. (2020). Follow-Up Studies of cf-DNA Testing from 101 Consecutive Fetuses and Related Ultrasound Findings. *Ultraschall Med*, *41*(2), 175-185.
67. Panchalee, T., Vossaert, L., Wang, Q., Crovetti, B. R., McCombs, A. K., Wapner, R. J., Van den Veyver, I. B., & Beaudet, A. L. (2020). The effect of maternal body mass index and gestational age on circulating trophoblast yield in cell-based noninvasive prenatal testing. *Prenat Diagn*, *40*(11), 1383-1389.
68. Cagino, K., Bortoletto, P., McCarter, K., Forlenza, K., Yau, A., Thomas, C., Melnick, A., & Prabhu, M. (2021). 980 Low fetal fraction in IVF pregnancies associated with hypertensive disorders of pregnancy [Conference Abstract]. *American Journal of Obstetrics and Gynecology*, *224*(2), S608.
69. Sapantzoglou, I., Gallardo Arozena, M., Dragoi, V., Akolekar, R., Nicolaides, K. H., & Syngelaki, A. (2021). Fetal fraction of cell free DNA in screening for hypertensive disorders at 11–13 weeks [Article]. *Journal of Maternal-Fetal and Neonatal Medicine*.
70. Suzumori, N., Sekizawa, A., Takeda, E., Samura, O., Sasaki, A., Akaishi, R., Wada, S., Hamanoue, H., Hirahara, F., Sawai, H., Nakamura, H., Yamada, T., Miura, K., Masuzaki, H., Nakayama, S., Kamei, Y., Namba, A., Murotsuki, J., Yamaguchi, M., . . . Sago, H. (2021). Retrospective details of false-positive and false-negative results in non-invasive prenatal testing for fetal trisomies 21, 18 and 13 [Article]. *European Journal of Obstetrics and Gynecology and Reproductive Biology*, *256*, 75-81.
71. Chung, E., & Pierce, B. (2020). Cell-free DNA fetal fraction and pregnancy outcome. *American Journal of Obstetrics and Gynecology*, *222*(1), S157-S157.
72. Clapp, M. A., Berry, M., Shook, L. L., Roberts, P. S., Goldfarb, I. T., & Bernstein, S. N. (2020). Low Fetal Fraction and Birth Weight in Women with Negative First-Trimester Cell-Free DNA Screening. *Am J Perinatol*, *37*(1), 86-91.
73. Clarence, A., Dekker, G., Arstall, M., Lee, E., Grehan, S., Jordan, A., Hall, J., Harraway, J., & Suthers, G. (2020). Impact of maternal exercise on the fraction of fetal cell free DNA in maternal plasma. *Pathology*, *52*, S107-S108.
74. Clausen, F. B., Barrett, A. N., Advani, H. V., Choolani, M., & Dziegiel, M. H. (2020). Impact of long‐term storage of plasma and cell‐free DNA on measured DNA quantity and fetal fraction. *Vox sanguinis*, *115*(7), 586-594.
75. Dai, R., Yu, Y., Zhang, H., Li, L., Jiang, Y., Liu, R., & Zhang, H. (2021). Analysis of 17,428 pregnant women undergoing non-invasive prenatal testing for fetal chromosome in Northeast China. *Medicine (Baltimore)*, *100*(6), e24740.
76. Dar, P., Jacobsson, B., MacPherson, C., Malone, F. D., Wapner, R. J., Roman, A., Khalil, A., Faro, R., Madankumar, R., Edwards, L., Haeri, S., Silver, R. M., Vohra, N., Hyett, J., Clunie, G., Demko, Z., Rabinowitz, M., Hakonarson, H., & Norton, M. E. (2021). 62 Multicenter prospective study of SNP-based cfDNA screening for aneuploidy with genetic confirmation in 18,496 pregnancies [Conference Abstract]. *American Journal of Obstetrics and Gynecology*, *224*(2), S44.
77. Dinsmoor, M., Endres, L., Van Den Berg, M., Maier, C., Yared, E., Lapin, B., & Plunkett, B. (2015). Obesity increases the risk for failure of non-invasive prenatal screening (NIPS), regardless of gestational age [Conference Abstract]. *American Journal of Obstetrics and Gynecology*, *212*(1), S401-S402.
78. Dolatkhah, M., Farzami, M. R., Khavari-Nejad, R. A., & Noori, S. (2021). Correlation of Maternal Age, Weight, Pregnancy-associated Plasma Protein A, Free Beta-human Chorionic Gonadotropin, Fetal Crown-rump Length, and Fetal Gender with Fetal DNA Fraction in Non-invasive Prenatal Testing: An Experiment on Iranian Pregnant Women. *Iranian Journal of Neonatology*, *12*(1), 26-32
79. Duvillier, C., Quibel, T., Felsenheld, C., Hupin-Genty, L., Cohen, C., & Vialard, F. (2019). [Cell-free DNA fraction: Can it be used to evaluate the risk of obstetrical issues?]. *Gynecol Obstet Fertil Senol*, *47*(11), 790-796.
80. Eiben, B., Krapp, M., Borth, H., Kutur, N., Kreiselmaier, P., Glaubitz, R., Deutinger, J., & Merz, E. (2015). Single Nucleotide Polymorphism-Based Analysis of Cell-Free Fetal DNA in 3000 Cases from Germany and Austria. *Ultrasound Int Open*, *1*(1), E8-e11
81. Galeva, S., Gil, M. M., Konstantinidou, L., Akolekar, R., & Nicolaides, K. H. (2019). First-trimester screening for trisomies by cfDNA testing of maternal blood in singleton and twin pregnancies: factors affecting test failure [Article]. *Ultrasound in obstetrics & gynecology : the official journal of the International Society of Ultrasound in Obstetrics and Gynecology*, *53*(6), 804-809.
82. Gerson, K. D., Truong, S., Haviland, M. J., O'Brien, B. M., Hacker, M. R., & Spiel, M. H. (2019). Low fetal fraction of cell-free DNA predicts placental dysfunction and hypertensive disease in pregnancy [Article]. *Pregnancy Hypertension*, *16*, 148-153.
83. Gil, M. M., Brik, M., Casanova, C., Martin-Alonso, R., Verdejo, M., Ramírez, E., & Santacruz, B. (2017). Screening for trisomies 21 and 18 in a Spanish public hospital: from the combined test to the cell-free DNA test. *J Matern Fetal Neonatal Med*, *30*(20), 2476-248
84. Giouzeppos, O., Flowers, N., Shi, G., Hunt, C., Scarff, K., Archibald, A., Norris, F., & Pertile, M. D. (2020). Post zygotic origin of trisomy as a cause of false negative NIPT results [Conference Abstract]. *Prenatal Diagnosis*, *40*, 64-65.
85. Guo, F. F., Yang, J. X., Huang, Y. L., Qi, Y. M., Hou, Y. P., Peng, H. S., Wang, D. M., Wang, Y. X., Luo, X. H., Li, Y., & Yin, A. H. (2019). Association between fetal fraction at the second trimester and subsequent spontaneous preterm birth. *Prenat Diagn*, *39*(13), 1191-1197
86. Guy, G. P., Hargrave, J., Dunn, R., Price, K., Short, J., Thilaganathan, B., & Collaborative, S. T. (2021). Secondary non-invasive prenatal screening for fetal trisomy: an effectiveness study in a public health setting. *Bjog-an International Journal of Obstetrics and Gynaecology*, *128*(2), 440-446.
87. Chen, F., Tan, M., Xu, Y., Zhu, B., Li, J., Lin, K., Chen, M., & Zeng, L. (2020). Retrospective analysis and mining of data from 10 840 patients undergoing non-invasive prenatal screening [Article]. *Zhonghua yi xue yi chuan xue za zhi = Zhonghua yixue yichuanxue zazhi = Chinese journal of medical genetics*, *37*(10), 1074-1078.
88. Hancock, S., Ben-Shachar, R., Adusei, C., Haverty, C., & Muzzey, D. (2020). Avoiding unnecessary trade-offs: Clinical experience for a noninvasive prenatal screen with both low no-call rate and high accuracy [Conference Abstract]. *Prenatal Diagnosis*, *40*, 66-68.
89. Hancock, S., Ben-Shachar, R., Adusei, C., Oyolu, C. B., Evans, E. A., Kang, H. P., Haverty, C., & Muzzey, D. (2020). Clinical experience across the fetal-fraction spectrum of a non-invasive prenatal screening approach with low test-failure rate [Article]. *Ultrasound in obstetrics & gynecology : the official journal of the International Society of Ultrasound in Obstetrics and Gynecology*, *56*(3), 422-430.
90. Hestand, M. S., Bessem, M., van Rijn, P., de Menezes, R. X., Sie, D., Bakker, I., Boon, E. M. J., Sistermans, E. A., & Weiss, M. M. (2019). Fetal fraction evaluation in non-invasive prenatal screening (NIPS) [Article]. *European Journal of Human Genetics*, *27*(2), 198-202.
91. Hopkins, M., Koelper, N., Burns, W., Durnwald, C., Sammel, M., & Dugoff, L. (2019). Cell-free DNA fetal fraction and risk for gestational diabetes. *American Journal of Obstetrics and Gynecology*, *220*(1), S584-S584.
92. Hopkins, M. K., Dugoff, L., Durnwald, C., Havrilesky, L. J., & Dotters-Katz, S. (2020). Cell-free DNA for Down syndrome screening in obese women: Is it a cost-effective strategy? [Article]. *Prenatal Diagnosis*, *40*(2), 173-178.
93. Hopkins, M. K., Koelper, N., Bender, W., Durnwald, C., Sammel, M., & Dugoff, L. (2020). Association between cell‐free DNA fetal fraction and gestational diabetes. *Prenatal diagnosis*, *40*(6), 724-727.
94. Hou, Y., Yang, J., Qi, Y., Guo, F., Peng, H., Wang, D., Wang, Y., Luo, X., Li, Y., & Yin, A. (2019). Factors affecting cell-free DNA fetal fraction: statistical analysis of 13,661 maternal plasmas for non-invasive prenatal screening. *Hum Genomics*, *13*(1), 6
95. Hu, H. J., Lee, M. Y., Cho, D. Y., Oh, M., Kwon, Y. J., Han, Y. J., Ryu, H. M., Kim, Y. N., & Won, H. S. (2020). Prospective clinical evaluation of Momguard non-invasive prenatal test in 1011 Korean high-risk pregnant women. *J Obstet Gynaecol*, *40*(8), 1090-1095.
96. Hu, P., Liang, D., Chen, Y., Lin, Y., Qiao, F., Li, H., Wang, T., Peng, C., Luo, D., & Liu, H. (2019). An enrichment method to increase cell-free fetal DNA fraction and significantly reduce false negatives and test failures for non-invasive prenatal screening: a feasibility study. *Journal of translational medicine*, *17*(1), 1-9.
97. Jelcic, D., Podobnik Brlečić, P., Podobnik, M., Kurdija, K., & Gebauer Vuković, B. (2019). Non invasive prenatal testing (NIPT) for common chromosomal aneuploidies data from a single center in a routine screening population [Conference Abstract]. *Journal of Perinatal Medicine*, *47*, eA448
98. Lapaire, O., Volgmann, T., Grill, S., Hosli, I., Zanetti-Daellenbach, R., Zhong, X. Y., & Holzgreve, W. (2009). Significant Correlation Between Maternal Body Mass Index at Delivery and in the Second Trimester, and Second Trimester Circulating Total Cell-free DNA Levels. *Reproductive Sciences*, *16*(3), 274-279.
99. Lázár, L., Nagy, B., Molvarec, A., & Rigó, J., Jr. (2010). [Quantity of total cell free and cell free fetal DNA in pregnancies with no complications and with preeclampsia]. *Orv Hetil*, *151*(19), 784-787.
100. Lazar, L., Rigó Jr, J., Nagy, B., Balogh, K., Makó, V., Cervenak, L., Mézes, M., Prohászka, Z., & Molvarec, A. (2009). Relationship of circulating cell-free DNA levels to cell-free fetal DNA levels, clinical characteristics and laboratory parameters in preeclampsia [Article]. *BMC Medical Genetics*, *10*, Article 120.
101. Li, J., Yuan, P., Wang, X., Tian, C., Chang, L., Gong, X., Ren, K., Wei, Y., & Zhao, Y. (2020). Influencing factors and pregnancy outcomes of unsuccessful cell-free DNA testing in maternal perinatal blood [Article]. *Chinese Journal of Perinatal Medicine*, *23*(9), 585-593
102. Liang, D., Lin, Y., Li, H., Hu, P., & Xu, Z. (2020). Analysis of follow-up information and pregnancy outcomes of cell free DNA prenatal screening. *Zhonghua fu Chan ke za zhi*, *55*(2), 106-111.
103. Lin, Y., Liang, D., Wang, Y., Li, H., Liu, A., Hu, P., & Xu, Z. F. (2020). Analyzing false-negative results detected in low-risk non-invasive prenatal screening cases. *Molecular Genetics & Genomic Medicine*, *8*(4), Article e1185.
104. Livergood, M. C., LeChien, K. A., & Trudell, A. S. (2017). Obesity and cell-free DNA "no calls": is there an optimal gestational age at time of sampling? *Am J Obstet Gynecol*, *216*(4), 413.e411-413.e419.
105. Lopes, J. L., Lopes, G. S., Enninga, E. A., Kearney, H. M., Hoppman, N. L., & Rowsey, R. A. (2020). Most noninvasive prenatal screens failing due to inadequate fetal cell free DNA are negative for trisomy when repeated. *Prenatal diagnosis*, *40*(7), 831-837.
106. Lu, Z., Tian, L., Ying, H., & Huang, F. (2020). Analysis of non-invasive prenatal testing in 14 047 cases of advanced age pregnant women [Article]. *Zhonghua yi xue yi chuan xue za zhi = Zhonghua yixue yichuanxue zazhi = Chinese journal of medical genetics*, *37*(6), 613-616.
107. Luo, Y., Hu, H., Jiang, L., Ma, Y., Zhang, R., Xu, J., Pan, Y., Long, Y., Yao, H., & Liang, Z. (2020). A retrospective analysis the clinic data and follow-up of non-invasive prenatal test in detection of fetal chromosomal aneuploidy in more than 40,000 cases in a single prenatal diagnosis center [Article]. *European Journal of Medical Genetics*, *63*(9).
108. Luo, Y., Hu, H., Zhang, R., Pan, Y., Ma, Y., Long, Y., Xu, J., Xu, L., Hu, B., Yao, H., & Chang, Q. (2020). Factors affecting the failure of non-invasive prenatal testing and the feasibility analysis of retesting [Article]. *Zhonghua yi xue yi chuan xue za zhi = Zhonghua yixue yichuanxue zazhi = Chinese journal of medical genetics*, *37*(6), 603-608.
109. Maiz, N., Alzola, I., Murua, E. J., & Rodríguez Santos, J. (2016). Cell-free DNA testing after combined test: factors affecting the uptake. *J Matern Fetal Neonatal Med*, *29*(21), 3558-3562
110. McKanna, T., Ryan, A., Krinshpun, S., Kareht, S., Marchand, K., Grabarits, C., Ali, M., McElheny, A., Gardiner, K., & LeChien, K. (2019). Fetal fraction‐based risk algorithm for non‐invasive prenatal testing: screening for trisomies 13 and 18 and triploidy in women with low cell‐free fetal DNA. *Ultrasound in Obstetrics & Gynecology*, *53*(1), 73-79.
111. Mesoraca, A., Margiotti, K., Dello Russo, C., Cesta, A., Cima, A., Longo, S. A., Barone, M. A., Viola, A., Sparacino, D., & Giorlandino, C. (2020). Cell-free DNA screening for aneuploidies in 7113 pregnancies: single Italian centre study. *Genet Res (Camb)*, *102*, e5.
112. Motevasselian, M., Saleh Gargari, S., Younesi, S., Pooransari, P., Saadati, P., Mirzamoradi, M., Savad, S., Taheri Amin, M. M., Modarresi, M. H., Afrakhteh, M., & Ghafouri-Fard, S. (2020). Non-invasive prenatal test to screen common trisomies in twin pregnancies [Article]. *Molecular Cytogenetics*, *13*(1)
113. Muzzey, D., Goldberg, J. D., & Haverty, C. (2020). Noninvasive prenatal screening for patients with high body mass index: Evaluating the impact of a customized whole genome sequencing workflow on sensitivity and residual risk [Article]. *Prenatal Diagnosis*, *40*(3), 333-341
114. Niemchak, T., Adamski, C., Allen, R., Woods, S., & Monroe, T. (2020). 1057: Noninvasive prenatal screening for fetal aneuploidy in twin pregnancies: A clinical laboratory experience [Conference Abstract]. *American Journal of Obstetrics and Gynecology*, *222*(1), S65
115. Noh, J. J., Ryu, H. M., Oh, S. Y., Choi, S. J., Roh, C. R., & Kim, J. H. (2019). A two-year experience of non-invasive prenatal testing (NIPT) at an urban tertiary medical center in South Korea. *Taiwan J Obstet Gynecol*, *58*(4), 545-551.
116. Qiu, Y., & Liu, C. (2019). Quantitative detection of cell-free fetal DNA in peripheral blood of pregnant women during early pregnancy. *Clinical and Experimental Obstetrics & Gynecology*, *46*(4), 611-614
117. Raynova, R., Bichev, S., Andonova, S., Yaneva, N., Kercheva, C., Bradinova, I., & Savov, A. (2019). Pilot study of locally performed noninvasive prenatal testing NIPT in Bulgaria [Conference Abstract]. *European Journal of Human Genetics*, *27*, 1196.
118. Rico, I. V., Salas, P. C., Rosa, C. S., Escobar, R. G., Aguilar, M. R. T., Alba, P. C., & Leon-Justel, A. (2019). Cell-free DNA analysis for trisomy 21 in first-trimester twin pregnancies. *Clinica Chimica Acta*, *493*, S596-S596.
119. Rodriguez, M., Reeder, C., Sylvester, K., Silva, L., & Genc, M. (2021). 1053 Abnormal placentation and affect on fetal fraction of cell-free DNA. *American Journal of Obstetrics & Gynecology*, *224*(2), S652
120. Schmid, M., Chen, K., White, K., Wang, Y., Doshi, J., & Kunz, L. (2019). OP05. 03: Changes in fetal cell‐free DNA fraction between consecutive maternal plasma samples in individual pregnancies. *Ultrasound in Obstetrics & Gynecology*, *54*, 99-99.
121. Scott, F. P., Menezes, M., Palma-Dias, R., Nisbet, D., Schluter, P., Costa, F. D., & McLennan, A. C. (2018). Factors affecting cell-free DNA fetal fraction and the consequences for test accuracy. *Journal of Maternal-Fetal & Neonatal Medicine*, *31*(14), 1865-1872.
122. Shani, H., Goldwaser, T., Keating, J., & Klugman, S. (2016). Chromosomal abnormalities not currently detected by cell-free fetal DNA: a retrospective analysis at a single center. *Am J Obstet Gynecol*, *214*(6), 729.e721-729.e711
123. Shani, H., Goldwaser, T., Keating, J., & Klugman, S. (2016). Chromosomal abnormalities not currently detected by cell-free fetal DNA: a retrospective analysis at a single center. *Am J Obstet Gynecol*, *214*(6), 729.e721-729.e711
124. Struble, C., Wang, E., Sparks, A., Song, K., Oliphant, A., & Cuckle, H. (2012). Analysis of maternal age, gestational age and aneuploidy on fraction of fetal cell-free DNA: P1-64. *Prenatal Diagnosis*, *32*.
125. Thurik, F. F., Lamain-de Ruiter, M., Javadi, A., Kwee, A., Woortmeijer, H., Page-Christiaens, G., Franx, A., van der Schoot, C. E., & Koster, M. P. H. (2016). Absolute first trimester cell-free DNA levels and their associations with adverse pregnancy outcomes. *Prenatal Diagnosis*, *36*(12), 1104-111
126. Urato, A. C., Peter, I., Canick, J., Lambert-Messerlian, G., Pulkkinen, A., Knight, G., Jeong, Y. J., Johnson, K. L., & Bianchi, D. W. (2008). Smoking in pregnancy is associated with increased total maternal serum cell-free DNA levels [Article]. *Prenatal Diagnosis*, *28*(3), 186-190
127. Urato, A. C., Peter, I., Canick, J., Lambert-Messerlian, G., Pulkkinen, A., Knight, G., Jeong, Y. J., Johnson, K. L., & Bianchi, D. W. (2008). Smoking in pregnancy is associated with increased total maternal serum cell-free DNA levels [Article]. *Prenatal Diagnosis*, *28*(3), 186-190
128. Welker, N. C., Lee, A. K., Kjolby, R. A. S., Wan, H. Y., Theilmann, M. R., Jeon, D., Goldberg, J. D., Haas, K. R., Muzzey, D., & Chu, C. S. (2020). High-throughput fetal fraction amplification increases analytical performance of noninvasive prenatal screening. *Genet Med*.
129. White, K., Wang, Y., Kunz, L. H., & Schmid, M. (2019). Factors associated with obtaining results on repeat cell-free DNA testing in samples redrawn due to insufficient fetal fraction. *J Matern Fetal Neonatal Med*, 1-6.
130. White, K., Wang, Y., Kunz, L. H., & Schmid, M. (2020). Factors associated with obtaining results on repeat cell-free DNA testing in samples redrawn due to insufficient fetal fraction [Article]. *Journal of Maternal-Fetal and Neonatal Medicine*, *33*(23), 4010-4015.
131. Wu, X. Q., Li, Y., Xie, X. R., Su, L. J., Cai, M. Y., Lin, N., Du, S. R., Xu, L. P., & Huang, H. L. (2020). Experience from 551 Pregnancies with Noninvasive Prenatal Testing-Positive Results in a Tertiary Referral Center. *Journal of Molecular Diagnostics*, *22*(12), 1469-1475.
132. Xie, X. L., Li, F. G., Tan, W. H., Yin, W. G., Chen, F. Y., & Guo, X. Y. (2019). The Effect of Freezing on Non-invasive Prenatal Testing. *Scientific Reports*, *9*, Article 6962.
133. Yang, X., Guo, X., Zhong, J., Chen, Z., & Wu, S. (2019). [Noninvasive prenatal genetic testing in 6804 pregnant women aged less than 35 years with positive results in serum screening]. *Nan Fang Yi Ke Da Xue Xue Bao*, *39*(11), 1350-1356.
134. Yared, E., Dinsmoor, M. J., Endres, L. K., Vanden Berg, M. J., Maier Hoell, C. J., Lapin, B., & Plunkett, B. A. (2016). Obesity increases the risk of failure of noninvasive prenatal screening regardless of gestational age. *Am J Obstet Gynecol*, *215*(3), 370.e371-376.
135. Yuan, X., Zhou, L., Zhang, B., Wang, H., Yu, B., & Xu, J. (2020). Association between low fetal fraction of cell free DNA at the early second-trimester and adverse pregnancy outcomes [Article]. *Pregnancy Hypertension*, *22*, 101-108.
136. Yuan, X. S., Zhou, L. N., Zhang, B., Wang, H. Y., Jiang, J., & Yu, B. (2019). Early second-trimester plasma cell free DNA levels with subsequent risk of pregnancy complications. *Clinical Biochemistry*, *71*, 46-51.
137. Zheng, Y. Y., Wan, S. N., Dang, Y. H., Song, T. T., Chen, B. L., & Zhang, J. F. (2019). Non-invasive prenatal testing for detection of trisomy 13, 18, 21 and sex chromosome aneuploidies in 8594 cases. *Ginekologia Polska*, *90*(5), 270-273
138. Zhou, D., Liang, D., Lv, W., Tian, F., Song, Z., Zhang, J., Ren, Y., Chen, D., Zhao, J., Zhou, K., Hou, Y., Gao, Y., & Wu, L. (2013). Noninvasive prenatal testing in China: Results and learning from a clinical study of more than 50,000 pregnancies [Conference Abstract]. *Prenatal Diagnosis*, *33*, 1
139. Zhou, Y., Zhu, Z., Gao, Y., Yuan, Y., Guo, Y., Zhou, L., Liao, K., Wang, J., Du, B., Hou, Y., Chen, Z., Chen, F., Zhang, H., Yu, C., Zhao, L., Lau, T. K., Jiang, F., & Wang, W. (2015). Effects of Maternal and Fetal Characteristics on Cell-Free Fetal DNA Fraction in Maternal Plasma. *Reprod Sci*, *22*(11), 1429-1435
140. Zhu, H., Jin, X., Xu, Y., Zhang, W., Liu, X., Jin, J., Qian, Y., & Dong, M. (2021). Efficiency of non-invasive prenatal screening in pregnant women at advanced maternal age [Article]. *BMC Pregnancy and Childbirth*, *21*(1).
141. Al Nakib, M., Desbrière, R., Bonello, N., Bretelle, F., Boubli, L., Gabert, J., & Levy-Mozziconacci, A. (2009). Total and fetal cell-free DNA analysis in maternal blood as markers of placental insufficiency in intrauterine growth restriction. *Fetal Diagn Ther*, *26*(1), 24-28
142. Al Toukhi, S., Chitayat, D., Keunen, J., Roifman, M., Seaward, G., Windrim, R., Ryan, G., & Van Mieghem, T. (2019). Impact of introduction of noninvasive prenatal testing on uptake of genetic testing in fetuses with central nervous system anomalies. *Prenat Diagn*, *39*(7), 544-54
143. Al-Ibraheemi, Z., Lewis, D., King, L., Bimson, B., & Porat, N. (2016). 773: Is high maternal level of cell-free fetal DNA associated with adverse pregnancy outcome? *American Journal of Obstetrics & Gynecology*, *214*(1), S404-S405.
144. Al-Omary, H. L., Alawad, Z. M., & Husseini, B. (2019). Cell-free DNA as a clinical indicator in maternal blood [Article]. *Turkish Journal of Endocrinology and Metabolism*, *23*(3), 174-180.
145. Botezatu, I., Serdyuk, O., Potapova, G., Shelepov, V., Alechina, R., Molyaka, Y., Ananév, V., Bazin, I., Garin, A., Narimanov, M., Knysh, V., Melkonyan, H., Umansky, S., & Lichtenstein, A. (2000). Genetic analysis of DNA excreted in urine: a new approach for detecting specific genomic DNA sequences from cells dying in an organism. *Clin Chem*, *46*(8 Pt 1), 1078-1084.
146. Cherry, A. M., Akkari, Y. M., Barr, K. M., Kearney, H. M., Rose, N. C., South, S. T., Tepperberg, J. H., & Meck, J. M. (2017). Diagnostic cytogenetic testing following positive noninvasive prenatal screening results: a clinical laboratory practice resource of the American College of Medical Genetics and Genomics (ACMG). *Genet Med*, *19*(8), 845-850.
147. Chitty, L. S., Hudgins, L., & Norton, M. E. (2018). Current controversies in prenatal diagnosis 2: Cell-free DNA prenatal screening should be used to identify all chromosome abnormalities. *Prenat Diagn*, *38*(3), 160-16
148. Čonka, J., Konečná, B., Lauková, L., Vlková, B., & Celec, P. (2017). Fetal DNA does not induce preeclampsia-like symptoms when delivered in late pregnancy in the mouse. *Placenta*, *52*, 100-10
149. Coroleucǎ, C., Ionescu, C. A., Pleş, L., Dimitriu, M., Banacu, M., Popescu, I., & Şerbǎnescu, L. (2016). Non-invasive prenatal testing for rare chromosomal anomalies [Article]. *Gineco.eu*, *12*(4), 208-210.
150. Deans, Z., Hill, M., Chitty, L. S., & Lewis, C. (2013). Non-invasive prenatal testing for single gene disorders: exploring the ethics. *Eur J Hum Genet*, *21*(7), 713-718.
151. Devers, P. L., Cronister, A., Ormond, K. E., Facio, F., Brasington, C. K., & Flodman, P. (2013). Noninvasive prenatal testing/noninvasive prenatal diagnosis: the position of the National Society of Genetic Counselors. *J Genet Couns*, *22*(3), 291-295
152. Drury, S., Hill, M., & Chitty, L. S. (2016). Cell-Free Fetal DNA Testing for Prenatal Diagnosis. *Adv Clin Chem*, *76*, 1-35.
153. Du, Y., Lin, J., Lan, L., Dong, Y., Zhu, J., Jiang, W., Pan, X., Lu, Y., Li, D., & Wang, L. (2018). Detection of chromosome abnormalities using current noninvasive prenatal testing: A multi-center comparative study. *Biosci Trends*, *12*(3), 317-324
154. Evans, M. I., Wapner, R. J., & Berkowitz, R. L. (2016). Noninvasive prenatal screening or advanced diagnostic testing: caveat emptor [Article]. *American Journal of Obstetrics and Gynecology*, *215*(3), 298-305.
155. Fan, H. C., & Quake, S. R. (2010). Sensitivity of noninvasive prenatal detection of fetal aneuploidy from maternal plasma using shotgun sequencing is limited only by counting statistics. *PLoS One*, *5*(5), e10439.
156. Fauzdar, A. (2014). Non-invasive prenatal testing (NIPT): A better option for patients [Conference Abstract]. *Molecular Cytogenetics*, *7*.
157. Flowers, N., Shi, G., Giouzeppos, O., Love, C., Bruno, D., & Pertile, M. (2016). Noninvasive prenatal testing (NIPT) for screening pregnancies of balanced reciprocal translocation carriers [Conference Abstract]. *Twin Research and Human Genetics*, *19*(5), 532
158. Gil, M. M., Galeva, S., Jani, J., Konstantinidou, L., Akolekar, R., Plana, M. N., & Nicolaides, K. H. (2019). Screening for Trisomies by cfDNA Testing of Maternal Blood in Twin Pregnancy: Update of the Fetal Medicine Foundation Results and Meta-analysis [Note]. *Obstetrical and Gynecological Survey*, *74*(11), 627-629.
159. Gomez-Lopez, N., Romero, R., Schwenkel, G., Garcia-Flores, V., Panaitescu, B., Varrey, A., Ayoub, F., Hassan, S. S., & Phillippe, M. (2020). Cell-Free Fetal DNA Increases Prior to Labor at Term and in a Subset of Preterm Births. *Reprod Sci*, *27*(1), 218-232.
160. Gruber, A., Pacault, M., El Khattabi, L. A., Vaucouleur, N., Orhant, L., Bienvenu, T., Girodon, E., Vidaud, D., Leturcq, F., Costa, C., Letourneur, F., Anselem, O., Tsatsaris, V., Goffinet, F., Viot, G., Vidaud, M., & Nectoux, J. (2018). Non-invasive prenatal diagnosis of paternally inherited disorders from maternal plasma: detection of NF1 and CFTR mutations using droplet digital PCR. *Clin Chem Lab Med*, *56*(5), 728-738.
161. Guy, C., Haji-Sheikhi, F., Rowland, C. M., Anderson, B., Owen, R., Lacbawan, F. L., & Alagia, D. P. (2019). Prenatal cell-free DNA screening for fetal aneuploidy in pregnant women at average or high risk: Results from a large US clinical laboratory. *Mol Genet Genomic Med*, *7*(3), e545.
162. Hedriana, H., Leonard, S., Ryan, A., & Martin, K. (2018). Fetal fraction (FF) characterization by zygosity in twin gestation using single nucleotide polymorphism (SNP)-based noninvasive prenatal testing (NIPT) [Conference Abstract]. *Prenatal Diagnosis*, *38*, 7
163. Hudecova, I., & Chiu, R. W. (2017). Non-invasive prenatal diagnosis of thalassemias using maternal plasma cell free DNA. *Best Pract Res Clin Obstet Gynaecol*, *39*,
